# Identification of a novel antisense noncoding RNA, *ALID*, transcribed from the putative imprinting control region of marsupial *IGF2R*

**DOI:** 10.1186/s13072-018-0227-8

**Published:** 2018-09-29

**Authors:** Shunsuke Suzuki, Geoffrey Shaw, Marilyn B. Renfree

**Affiliations:** 1 Department of Agricultural and Life Sciences, Faculty of Agriculture, Shinshu University, Nagano, 399-4598 Japan; 2 Department of Interdisciplinary Genome Sciences and Cell Metabolism, Institute for Biomedical Sciences, ICCER, Shinshu University, Nagano, 399-4598 Japan; 3 School of BioSciences, The University of Melbourne, Victoria, 3010 Australia

**Keywords:** Genomic imprinting, *IGF2R*, lncRNA, Marsupials

## Abstract

**Background:**

Genomic imprinting leads to maternal expression of *IGF2R* in both mouse and opossum. In mouse, the antisense long noncoding (lnc) RNA *Airn,* which is paternally expressed from the differentially methylated region (DMR) in the second intron of *Igf2r,* is required to silence the paternal *Igf2r*. In opossum, however, intriguingly, the DMR was reported to be in a different downstream intron (intron 11) and there was no antisense lncRNA detected in previous analyses. Therefore, clarifying the imprinting mechanism of marsupial *IGF2R* is of great relevance for understanding the origin and evolution of genomic imprinting in the *IGF2R* locus. Thus, the antisense lncRNA associated with the marsupial DMR can be considered as the ‘missing link’. In this study, we identified a novel antisense lncRNA, *ALID,* after detailed analysis of the *IGF2R* locus in an Australian marsupial, the tammar wallaby, *Macropus eugenii*, and compared it to that of the grey short-tailed opossum, *Monodelphis domestica.*

**Results:**

Tammar *IGF2R* showed maternal expression and had a maternally methylated CpG island (CGI) in intron 12 as well as a promoter CGI without differential methylation, but none in the second intron. Re-analysis of the *IGF2R* of opossum detected the CGI in intron 12, not intron 11, as previously reported, confirming that the DMR in intron 12 is conserved between these marsupials and so is the putative imprinting control region of marsupial *IGF2R*. *ALID* is paternally expressed from the middle of the DMR and is approximately 650 bp long with a single exon structure that is extremely short compared to *Airn*. Hence, the lncRNA transcriptional overlap of the *IGF2R* promoter, which is essential for the *Igf2r* silencing in the mouse, is likely absent in tammar. This suggests that fundamental differences in the lncRNA-based silencing mechanisms evolved in eutherian and marsupial *IGF2R* and may reflect the lack of differential methylation in the promoter CGI of marsupial *IGF2R*.

**Conclusions:**

Our study thus provides the best candidate factor for establishing paternal silencing of marsupial *IGF2R* without transcriptional overlap, which is distinct from the *Igf2r* silencing mechanism of *Airn,* but which may be analogous to the mode of action for the flanking *Slc22a2* and *Slc22a3* gene silencing in the mouse placenta.

**Electronic supplementary material:**

The online version of this article (10.1186/s13072-018-0227-8) contains supplementary material, which is available to authorized users.

## Background

Genomic imprinting is an epigenetic mechanism which regulates parent-of-origin-dependent expression of imprinted genes. In higher vertebrates, it has been observed only in mammals and is limited to viviparous mammalian groups, the eutherians and marsupials [1–6] and appears to be absent in the egg-lying monotremes [7]. While more than 100 imprinted genes have been identified in eutherians so far, it has become clear that only small subset of them are also imprinted in marsupials [8–17]. Most imprinted genes are highly expressed in the placenta, and functions of imprinted genes are often associated with placental development and growth, foetal and postnatal development and growth, control of maternal behaviour related to postnatal care and also lactation [3–5, 18]. Therefore, the evolution of genomic imprinting might be correlated with the evolution of mammalian viviparity. The study the divergent evolutionary pathways taken by therian mammals of imprinting mechanism would contribute to understanding how mammals have acquired complex epigenomic regulation because genomic imprinting has been an excellent model revealing many epigenetic mechanisms to control gene expression.

The mouse *Igf2r* locus on chromosome 17 is one of the best characterised imprinted domains which includes the maternally expressed *Igf2r*, *Slc22a2* and *Slc22a3* genes and the paternally expressed lncRNA gene *Airn* [19, 20]. The *Airn* lncRNA is an established example of a cis-silencing lncRNA essential for the paternal silencing of *Igf2r*, *Slc22a2* and *Slc22a3* [21–24]*. Airn* is transcribed from the maternally methylated DMR located in the *Igf2r* intron 2 in antisense orientation against to *Igf2r* [25, 26]. *Airn* transcription overlaps the *Igf2r* promoter but not the *Slc22a2* or *Slc22a3* promoters. *Airn* transcriptional overlap of the *Igf2r* promoter, not its lncRNA products, is required for paternal silencing of *Igf2r*, interfering with RNA polymerase II recruitment to the *Igf2r* promoter [27]. On the other hand, paternal silencing of *Slc22a3* in the placenta depends on the *Airn* lncRNA products recruiting the H3K9 histone methyltransferase G9a to the *Slc22a3* promoter [28]. Thus, *Airn* has two different modes of action to silence paternal *Igf2r* and *Slc22a3*.

In humans, adult tissues lack *IGF2R* imprinted expression [29, 30], but it is found in foetal tissues, placenta, cultured amniotic cells, lymphoblastoid cells and Wilms’ tumours where it is polymorphic [31–34]. The human *IGF2R* intronic CpG island has promoter activity, and the human orthologue of *Airn* is present at least in Wilms’ tumours [35]. *IGF2R* shows imprinted expression also in sheep and dogs, but not in pigs [36–38]. Although the imprinted expression pattern of *IGF2R* is not identical in these eutherian species, the intron 2 CpG islands are maternally methylated equally.

In marsupials, there is maternal expression of *IGF2R* in the opossum, *Monodelphis domestica* [39]. However, interestingly, Das et al. [40] found a maternally methylated DMR in opossum *IGF2R* intron 11 (we argue that it is intron 12, see discussion) not intron 2 and there was no antisense lncRNA found around the opossum *IGF2R* DMR. Given the presence of marsupial *IGF2R* DMR at a different genomic location from that of the mouse, studying the imprinting control mechanism in the marsupial *IGF2R* locus would be of great importance to understand the evolution of genomic imprinting in mammals. Hence, the antisense lncRNA associated with the marsupial DMR can be considered as the ‘missing link’ to investigate imprinting mechanism of marsupial *IGF2R* unless there is an as yet unidentified lncRNA-independent mechanism that regulates marsupial *IGF2R* imprinting.

In this study, we demonstrate that tammar *IGF2R* is also maternally expressed and that the CGI located in tammar *IGF2R* intron 12 is a maternally methylated DMR. Furthermore, we identify a novel paternally expressed antisense lncRNA, *ALID* (Antisense LncRNA in the *IGF2R* DMR), which is transcribed from the middle of the tammar *IGF2R* DMR. Because of the extremely short 650 bp length of *ALID* compared to *Airn*, lncRNA transcriptional overlap of the *IGF2R* promoter, which is essential for the *Igf2r* silencing in the case of mouse, is likely absent in tammar. This suggests that there is fundamental difference in the lncRNA-based silencing mechanisms between eutherian and marsupial *IGF2R*. Our study thus provides the most likely candidate factor for establishing paternal silencing of marsupial *IGF2R* without transcriptional overlap, which is distinct from the *Igf2r* silencing mechanism of *Airn*, but may be analogous to mode of action for the flanking *Slc22a3* silencing in the mouse placenta.

## Results

### Maternal expression of tammar IGF2R

**Fig. 1:**
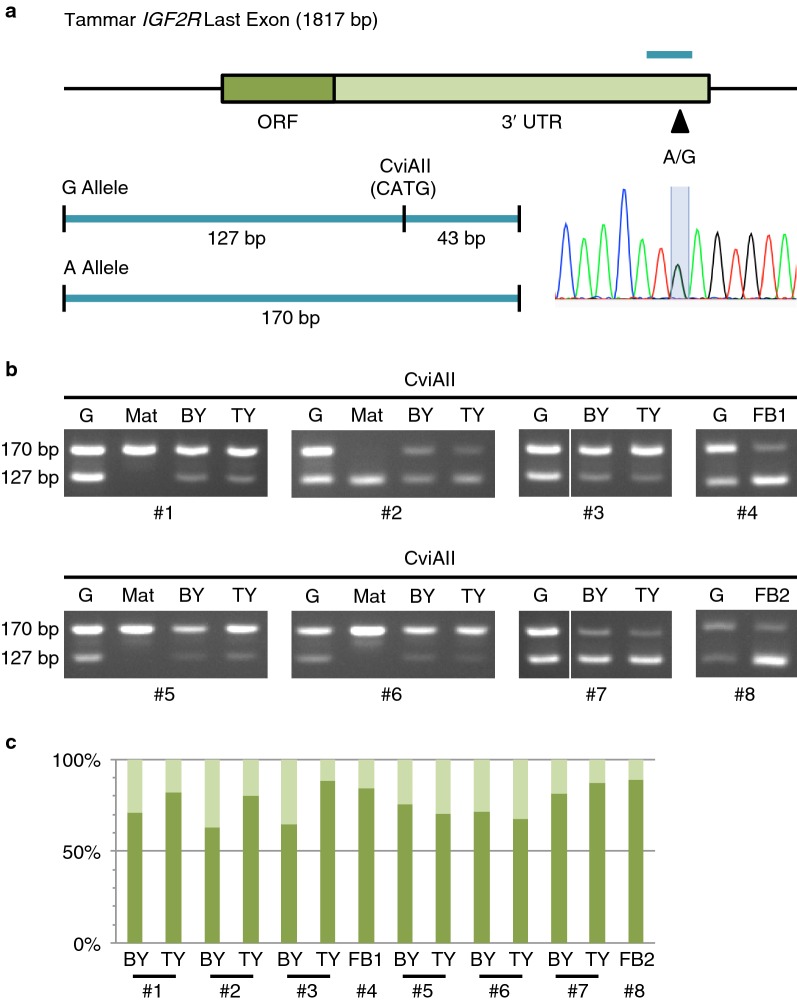
Imprinting analysis of tammar *IGF2R*. **a** Single nucleotide polymorphism in tammar *IGF2R*. The dark and light green parts in the box represent ORF and 3′ UTR of the last exon of tammar *IGF2R*, respectively. The black arrowhead and light blue line show the position of polymorphic site and the region of PCR amplification that were used for allelic expression analysis by RFLP analysis, respectively. The 170 bp PCR product contains a recognition sequence of CviAII restriction enzyme for G allele but not for A allele, enabling RFLP analysis. The sequencing data of a heterozygous sample show double peak of A and G at the polymorphic site. **b** Allelic expression pattern of tammar *IGF2R*. The intensity of 170 and 127 bp bands shows expression level from A allele and G allele, respectively. *G* genomic DNA, *Mat* maternal genotype, *BY* yolk sac placenta (bilaminar region), *TY* yolk sac placenta (trilaminar region), *FB* fibroblast cell line, #; individual number. **c** Allelic expression ratio of tammar *IGF2R*. The dark and light green bars represent expression ratio of active and inactive alleles, respectively. Each ratio was calculated under the normalisation using genomic DNA data as 50%

We first searched for polymorphism in the 3′ untranslated region (UTR) of tammar *IGF2R* to distinguish paternal and maternal alleles. Only single nucleotide polymorphism (SNP) site was found at the very end of the last exon with A/G sequence variation (Fig. 1a). This SNP enabled us to perform restriction fragment length polymorphism (RFLP) analysis by the presence or absence of the CviAII restriction recognition site (Fig. 1a). After CviAII digestion for the PCR products amplified from genomic DNA and cDNA of bilaminar and trilaminar regions of the yolk sac placentae and of fibroblast-like primary cell cultures as well as maternal DNA, the intensity of cut and uncut bands in gel electrophoresis was quantified. Expression of tammar *IGF2R* was clearly biased to either the A or G alleles that produce uncut and cut bands, respectively (Fig. 1b, c). In the 4 individuals, #1, 3, 5, 6, the A allele predominantly expressed *IGF2R* mRNA while in the other 4 cases, #2, 4, 7, 8, reciprocally biased expression was observed (Fig. 1b). Of these, the 4 informative cases for maternal genotype, #1, 2, 5, 6, showed that maternal allele was predominantly transcribed. These data demonstrated that tammar *IGF2R* is also a maternally expressed imprinted gene as in mouse and opossum.

### DNA methylation analysis of CpG islands in the promoter and intron 12 of tammar IGF2R

**Fig. 2:**
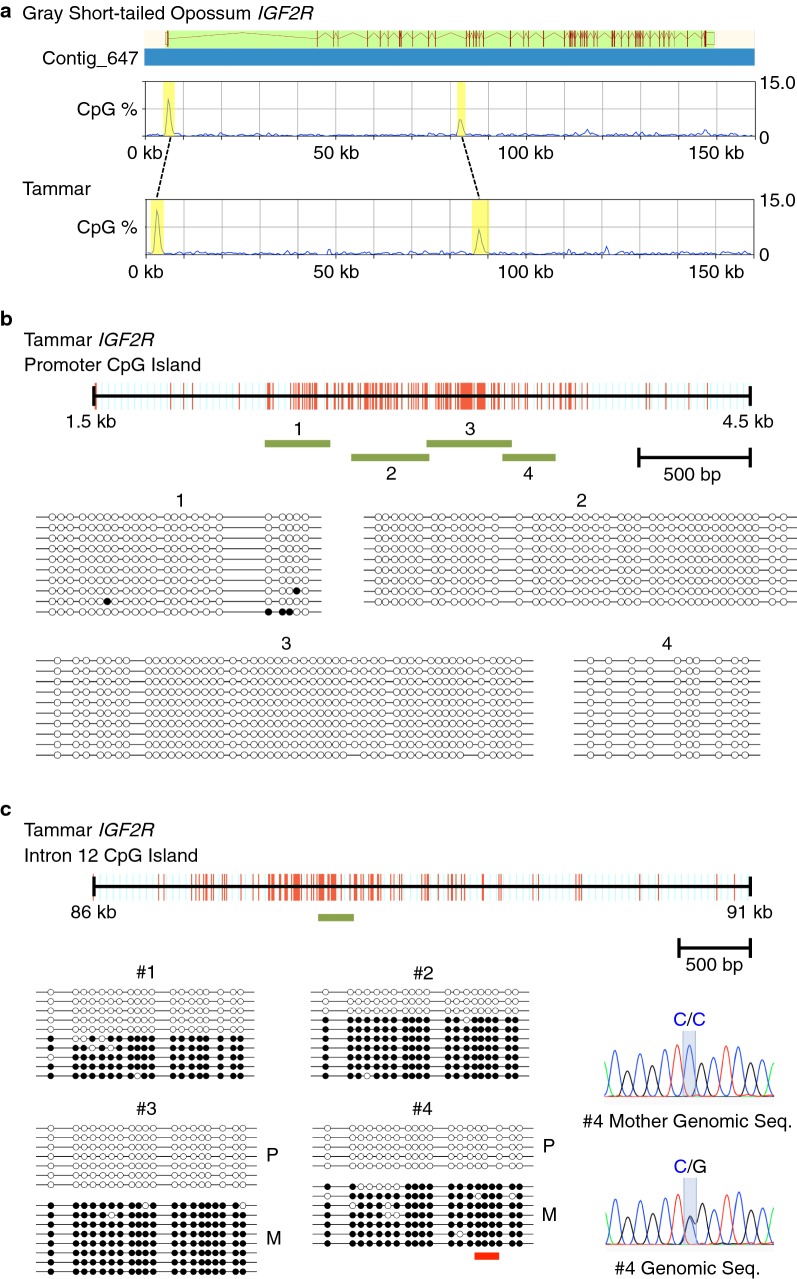
DNA methylation analysis in tammar *IGF2R*. **a** Location of CpG islands in opossum and tammar *IGF2R*. The graphs show the density of CpG sites in genomic sequences including opossum and tammar *IGF2R*, respectively. The yellow highlighted peaks in the graphs indicate the promoter and intron 12 CpG islands in opossum *IGF2R* and the orthologous CpG islands detected in tammar *IGF2R* genomic sequence. **b** DNA methylation analysis of promoter CpG island in tammar *IGF2R*. The red bars indicate each CpG site in 3 kb genomic sequence including the tammar *IGF2R* promoter CpG island. The green lines represent the regions analysed by bisulphite sequencing. Open circle; unmethylated CpG site, filled circle; methylated CpG site. **c** DNA methylation analysis of intron 12 CpG island in tammar *IGF2R*. The red line in #4 data represents the location of a single nucleotide polymorphism. Direct sequencing of PCR products amplified from the mother’s genomic DNA clarified maternal allele-specific DNA methylation

Given the imprinting of tammar *IGF2R*, we next analysed DNA methylation to investigate the possible imprinting mechanism of the *IGF2R* locus in marsupials. One CGI was located over the putative promoter region of tammar *IGF2R*, while the other CGI was found in intron 12 at an orthologous position to the opossum intron 11 CGI which forms a DMR (Fig. 2a). Bisulphite sequencing revealed that most CpG sites were unmethylated in almost the entire region of the promoter CGI, suggesting that differential epigenetic modification other than DNA methylation at the promoter region, such as histone modification, regulates maternal expression of tammar *IGF2R* (Fig. 2b). In contrast, clear differential methylation was observed in the intron 12 CGI (Fig. 2c). The two informative cases having a SNP inside the amplified region enabled us to determine that the methylated allele was maternally transmitted. Thus, it was demonstrated that the intron 12 CGI is a maternally methylated DMR and the putative imprinting control region of marsupial *IGF2R*.

### Detection and determination of the structure of ALID lncRNA

**Fig. 3:**
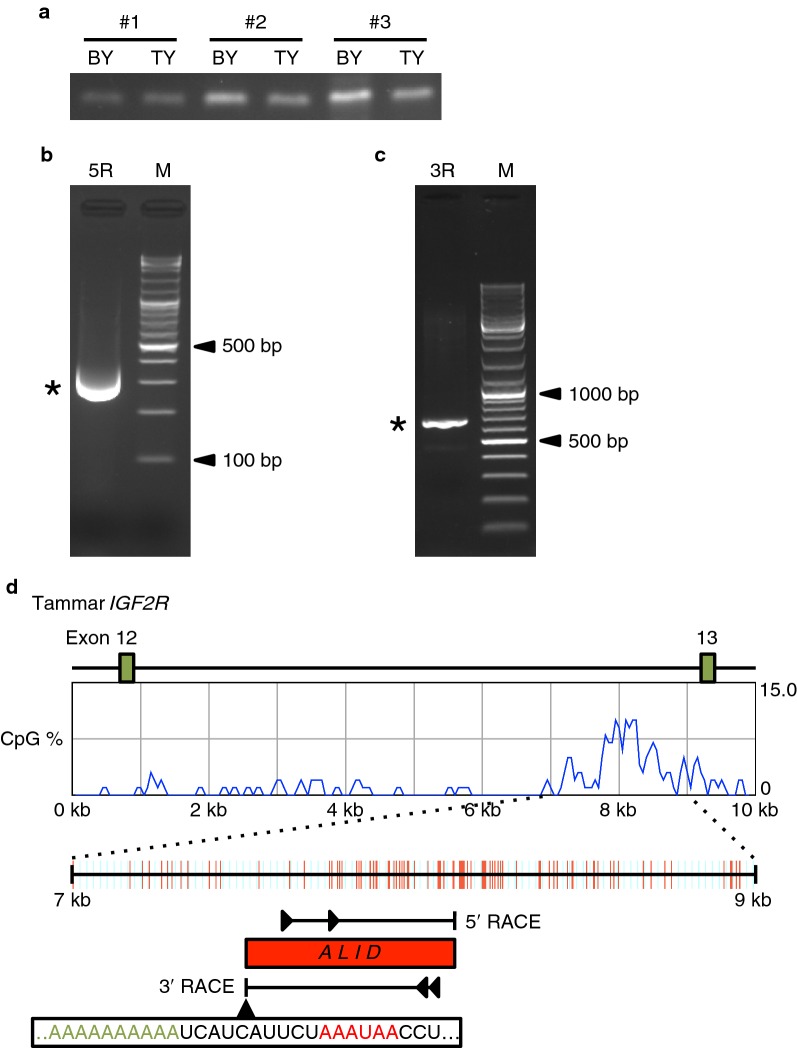
Determination of the transcript structure of *ALID*. **a** Strand-specific RT-PCR to detect antisense transcript nearby tammar *IGF2R* DMR. *BY* yolk sac placenta (bilaminar region), *TY* yolk sac placenta (trilaminar region). **b** Gel photograph of 5′ RACE experiment to determine transcription start site of *ALID*. **c** Gel photograph of 3′ RACE experiment to determine polyadenylation site of *ALID*. **d** Summary illustration for the structure of *ALID*. The graph shows the density of CpG sites in intron 12 sequence of tammar IGF2R. Two green boxes represent position of *IGF2R* exon 12 and 13 in the 10 kb genomic sequence, respectively. The red bars indicate each CpG site in 2 kb sequence around intron 12 CpG island. The arrowheads represent (nested) gene-specific primers used for 5′ and 3′ RACE experiments. The red box shows full-length *ALID* lncRNA revealed by RACE experiments. Canonical polyadenylation signal sequence (red) was found nearby polyadenylation site (green) of *ALID*

To compare the regulatory mechanisms of *IGF2R* imprinting between eutherians and marsupials, we examined whether there is any antisense lncRNA which is transcribed from intron 12 DMR similar to *Airn* of the mouse *Igf2r* locus. To selectively reverse transcribe antisense transcripts, strand-specific RT-PCR was performed using a reverse transcription primer designed at the upstream region of the DMR in intron 12 in sense orientation against *IGF2R* (Fig. 3a). The results indicated the presence of any antisense transcripts nearby the DMR, although expression level seemed not to be high in the yolk sac placenta. Hence we next carried out 5′ and 3′ RACE (rapid amplification of cDNA ends) to determine the full-length structure of this antisense transcript (Fig. 3b, c). Sequencing the nested 5′ RACE product, it appeared that the transcription start site (TSS) of the antisense transcript was located in the middle of the DMR, suggesting that this antisense transcript is the marsupial equivalent to murine *Airn* (Fig. 3d). Combining the data of 3′ RACE, it was revealed that this antisense transcript is approximately 650 bp long with a single exon structure and no obvious protein-coding potential. A typical polyadenylation signal was located before the polyA tail, suggesting the result of 3′ RACE was not an artefact (Fig. 3d). Thus, we named this antisense lncRNA *ALID*.

### Expression and imprinting analyses of ALID

**Fig. 4:**
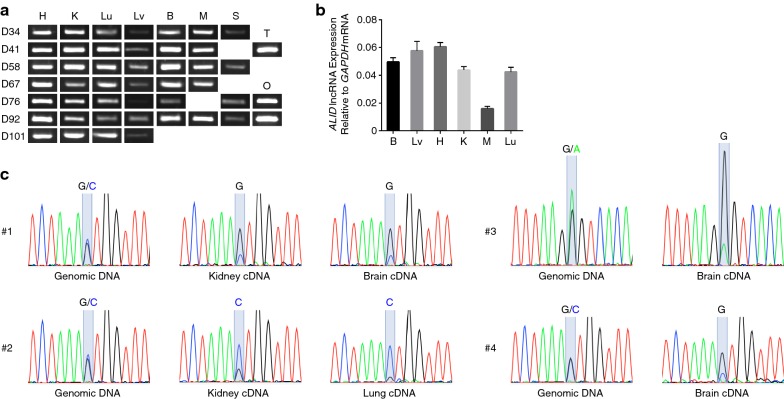
Expression analyses of *ALID*. **a** Expression analysis of *ALID* by strand-specific RT-PCR. The left labels indicate stages of pouch young animals (days from postpartum). *H* heart, *K* kidney, *Lu* lung, *Lv* liver, *B* brain, *M* skeletal muscle, *S* spleen, *T* testis, *O* ovary. **b** Quantification of expression level of *ALID* by strand-specific RT-qPCR. **c** Allelic expression analysis of *ALID* by direct sequencing followed by strand-specific RT-PCR. DNA methylation analysis of nearby genomic region including these SNP sites showed that the expressed alleles were unmethylated alleles (data not shown), demonstrating paternal expression of *ALID* lncRNA

We confirmed expression of *ALID* in a range of tissues from developing young by both conventional PCR and strand-specific reverse transcription followed by qPCR. *ALID* was ubiquitously expressed in various stages of tammar pouch young although there was some degree of difference in expression levels between tissues (Fig. 4a, b). The imprinting status of *ALID* was determined by direct sequencing of strand-specific RT-PCR products that contain a SNP site. In contrast to the data of genomic DNA PCR products in which double peaks at SNP site have almost the same signal strength, the data of RT-PCR products clearly showed that *ALID* is predominantly expressed from either one of the two alleles (Fig. 4c). Remarkably, in the data of individual #2, the expressed allele was opposite to the data of #1 and #4, showing that the monoallelic expression observed in these data was not consequence of simple allelic difference but reflected the parental origin. DNA methylation analysis of the nearby genomic region including these SNP sites showed that the expressed alleles were unmethylated alleles (data not shown), demonstrating the paternal expression of *ALID* lncRNA.

## Discussion

In this study, we identified a novel antisense lncRNA, *ALID*, analysing the *IGF2R* locus in an Australian marsupial, the tammar wallaby and compared it to that of opossum. We first clarified that both maternal expression and DMR of *IGF2R* were conserved between tammar and opossum. Confirming the evidence of the downstream DMR in *IGF2R* of an Australian marsupial is important to ensure the previous finding in opossum because the data of differential methylation was provided from only a single sample in the opossum study. According to the report by Das et al., the opossum *IGF2R* DMR was located in intron 11 [40]. However, the two exons neighbouring the tammar *IGF2R* DMR were exon 12 and 13 that are orthologous to exon 12 and 13 in both mouse and human *IGF2R*. Because all the orthologous exons of the main transcript of mouse and human *IGF2R* are found in both tammar and opossum genomic sequences and the tammar and opossum *IGF2R* DMRs were clearly located to the orthologous position, we conclude that the marsupial *IGF2R* DMR is present in intron 12 not 11.

While the downstream CGI in tammar *IGF2R* intron 12 was maternally methylated, the promoter CGI was entirely unmethylated despite maternal expression of tammar *IGF2R*. This observation provided good agreement with the previous work in opossum [40], suggesting that other differential epigenetic modification than DNA methylation such as differential histone marks on the *IGF2R* promoter region is responsible for the paternal silencing of marsupial *IGF2R*. In our allelic expression analyses, there is some level of leaky expression from the repressed paternal alleles detected in all the samples examined. This might be the result of repression of the paternal tammar *IGF2R* promoter by DNA methylation-independent epigenetic mechanism because similar leaky expression from the repressed allele was also observed in some imprinted genes in both marsupials and eutherians that are regulated by differential histone modifications without DNA methylation [9, 11, 41].

We also identified an antisense lncRNA transcribed from the middle of the tammar *IGF2R* DMR which is located in intron 12 and named it *ALID*. *ALID* was predominantly expressed from unmethylated alleles, indicating its paternal expression. Thus, despite the non-orthologous location of DMRs between mouse and tammar, *Airn* and *ALID* share a common feature that both are paternally expressed from the middle of DMRs. However, surprisingly, the length of *ALID* was extremely short ~ 650 bp, compared to *Airn* length which is more than 100 kb [26]. This result is consistent with the strand-specific RT-PCR experiments performed in the previous study in opossum which did not detect any antisense transcript, because the position of all the primers used in their strand-specific RT-PCR experiments was too far from the DMR to detect this short transcript [40]. We fortunately detected *ALID* in this study because the primer position was designed closer to the middle of DMR where we presumed that the transcription start site exists. The short length of *ALID* suggests certain critical differences exist in the regulatory mechanisms of *IGF2R* imprinting between mouse and tammar. Murine *Airn* transcriptionally overlaps with the *Igf2r* promoter and this transcriptional overlap, but not the lncRNA product itself, is crucial for paternal *Igf2r* silencing [27], while tammar *ALID* does not overlap with the *IGF2R* promoter due to its short length and the much more downstream intron 12 location of DMR, suggesting the marsupial *IGF2R* imprinting mechanism does not require the promoter overlap unlike the murine case. While the promoter overlap with *Airn* is critical for *Igf2r* imprinting, the two adjacent genes *Slc22a2* and *Slc22a3* that are imprinted only in the placenta do not require promoter overlap with *Airn* [42]. It is thus possible that the mechanism regulating marsupial *IGF2R* imprinting is similar to the promoter overlap-independent silencing mechanism regulating imprinting of *Slc22a2* and *Slc22a3* in the mouse placenta. Alternatively, there is a possibility that an as yet unknown very long form of *ALID* is expressed only in a certain developmental window in early embryonic stages and makes transcriptional overlap with *IGF2R* promoter. After the establishment of epigenetic silencing of paternal *IGF2R*, the structure of *ALID* may be changed to the short form, which would be functionless in this case.

**Fig. 5:**
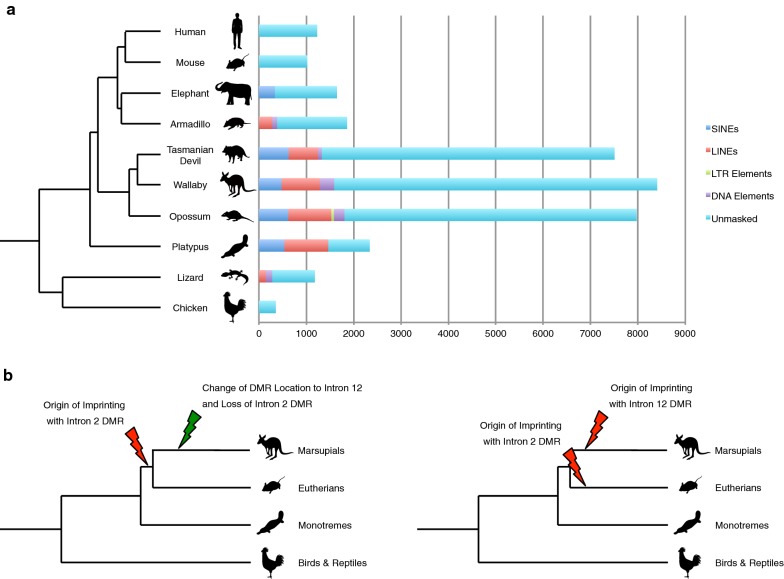
Evolution of intron 12 length and models for the origin of imprinting in *IGF2R*. **a** The marsupial-specific elongation of intron 12 length in *IGF2R*. Length of the bars represents *IGF2R* intron 12 lengths in each species. The multiple colours in the bars show rates of transposable elements in intron 12 sequences that were calculated by RepeatMasker (www.repeatmasker.org). Blue; SINEs, Red; LINEs, Green; LTR elements, Purple; DNA elements, Light blue; unmasked unique sequences. **b** Two hypotheses for the origin of imprinting in *IGF2R*. Left; the origin of imprinting of the *IGF2R* locus was just the once in the common ancestor of eutherians and marsupials, and the change of DMR location to intron 12 and consequent loss of former DMR in intron 2 have been occurred in the marsupial ancestor. Right; the acquisition of *IGF2R* imprinting has been independently occurred in both eutherian ancestor and marsupial ancestor

Interestingly, we found that the length of the *IGF2R* intron 12 has been largely extended specifically in marsupial species (Fig. 5a), while no such clear difference was observed for the intron 2 lengths among multiple species. This phenomenon is most likely linked to the marsupial-specific acquisition of the intron 12 DMR. Hence, it is simple to hypothesise that the intron 12 DMR emerged in the common ancestor of marsupials rather than that it has occurred in the common therian ancestor and only eutherians have lost it after the divergence of marsupials. Then, was the emergence of the intron 12 DMR de novo acquisition of imprinting to *IGF2R* or not? This question is important to discuss if the *IGF2R* locus had two independent origins of imprinting or just the once. We provide two simple models for the evolution of *IGF2R* imprinting as it is difficult to conclude the most likely hypothesis currently (Fig. 5b). The first possibility is based on the idea that the origin of imprinting of the *IGF2R* locus occurred just once in the common therian ancestor while the change of DMR location to intron 12 and consequent loss of former DMR in intron 2 subsequently occurred in the marsupial ancestor (Fig. 5b left). Alternatively, the acquisition of *IGF2R* imprinting might have independently arisen separately in both the eutherian and marsupial ancestors (Fig. 5b right).

## Conclusions

In this study, we provide the most likely lncRNA candidate factor for establishing paternal silencing of marsupial *IGF2R* without transcriptional overlap, which is distinct from the *Igf2r* silencing mechanism of *Airn,* but may be analogous to the mode of action for the flanking *Slc22a2* and *Slc22a3* silencing that occurs in the mouse placenta. Although we clarified that a novel DMR in intron 12 emerged in the common ancestor of marsupials, it is still unclear whether this event was due to a change of DMR location or de novo acquisition of *IGF2R* imprinting. Both scenarios represent the extraordinary history of this imprinted locus. The former scenario would provide the first evidence of the change of DMR location in any imprinted domains, and the latter scenario would be the first example of two independent origins of genomic imprinting for the same gene, suggesting extremely strong positive selection for *IGF2R* imprinting during mammalian evolution.

## Methods

### Animals and tissue collection

Tammar wallabies of Kangaroo Island origin were maintained in our breeding colony in grassy, outdoor enclosures. Lucerne cubes, grass and water were provided ad libitum and supplemented with fresh vegetables. Yolk sac placenta tissues were collected between days 21 and 26 of the 26.5 days gestation as previously described [43, 44]. Pouch young between days 34 and 101 postpartum were dissected to obtain a range of tissues. Experimental procedures conformed to Australian National Health and Medical Research Council (2013) guidelines and were approved by the Animal Experimentation Ethics Committees of the University of Melbourne.

### RFLP analysis

The 3′ UTR of *IGF2R*, including the polymorphism on the CviAII recognition sequence, was amplified by 35 cycles of RT-PCR. The PCR products were digested with 1–5 units of CviAII for 3 h at 25 °C. The digested PCR products were resolved by gel electrophoresis. The intensity of the cut and uncut bands was quantified with ATTO CS Analyzer 3 software (ATTO).  Primer sequences used in this study are provided in the Additional file 1.

### Bisulphite sequencing

Genomic DNA was extracted from yolk sac placentas using Trizol (Life Technologies). Genomic DNA was treated with sodium bisulphite solution, as described previously [45, 46]. After the bisulphite treatment of the genomic DNA, 35 cycles of PCR were carried out using the primers designed by MethPrimer [47]. The PCR products were cloned using a pTAC-2 vector (BioDynamics Laboratory) and ECOS-competent *Escherichia coli* DH5α (NIPPON GENE). Plasmids were purified using FastGene Plasmid Mini (NIPPON Genetics) and sequenced. The sequence data were analysed by QUMA (quantification tool for methylation analysis; http//quma.cdb.riken.jp) [48].  Primer sequences used in this study are provided in the Additional file 1.

### Strand-specific RT-PCR

Total RNA was extracted from yolk sac placentas and a panel of pouch young tissues using Trizol (Life Technologies) as instructed by the manufacturer. Total RNA was treated with DNase I to remove genomic DNA (Promega, M6101). cDNA was synthesised using a Transcriptor First Strand cDNA Synthesis Kit (Roche) with the primer designed in the intron 12 DMR in sense direction for *IGF2R.* There was no amplification from the minus RT controls detected for any of the RNA samples examined in this study. Thirty-five cycles of PCR amplification were carried out in 10 µl total volume with 10 ng cDNA using 0.2 U TaKaRa Ex Taq HS (TaKaRa), 4 pmol of each primer and 2 nmol of each dNTP mixture under the following cycle condition: 96 °C for 15 s, 60 °C for 30 s and 72 °C for 30 s. PCR products were resolved by gel electrophoreses. Primer sequences used in this study are provided in the Additional file 1.

### 5′ and 3′ RACE experiments

To determine the complete structure of *ALID*, we performed 5′ and 3′ RACE experiments using a SMARTer RACE 5′/3′ Kit (Clontech) according to the manufacturer’s instructions. The RACE products amplified from the templates created using pouch young brain RNA were cloned using a pTAC-2 vector (BioDynamics Laboratory) and ECOS-competent *Escherichia coli* DH5α (NIPPON GENE). Plasmids were purified using FastGene Plasmid Mini (NIPPON Genetics) and sequenced. Primer sequences used in this study are provided in the Additional file 1.

### Quantitative RT-PCR

cDNA was prepared as described in the strand-specific RT-PCR section. Quantitative real-time polymerase chain reaction (qPCR) was carried out in triplicate in 10 µl volumes containing 25 ng cDNA, 5 nM of the primers and FastStart Essential DNA Green Master (Roche) using LightCycler 96 (Roche). The amplification efficiency was calculated from the standard curve. GAPDH was used as the reference gene, and the data were analysed by Microsoft Excel.  Primer sequences used in this study are provided in the Additional file 1.

## Additional file

**Additional file 1.** List of primer sequences.
