# Supplementary material for: Identification of a novel antisense noncoding RNA, ALID, transcribed from the putative imprinting control region of marsupial IGF2R
Source: Epigenetics Chromatin. 2018 Sep 29;11:55. doi: 10.1186/s13072-018-0227-8 (PMC6162910; doi:10.1186/s13072-018-0227-8)
Supplement: Supplementary file 1 — Additional file 1. List of primer sequences. [file 13072_2018_227_MOESM1_ESM.docx]

**Supplementary Material: List of primer sequences**

DNA methylation analysis in the intron 12 CpG island

F; 5'- TTTTAAAGTTTTTTTAAAAGGGTAA -3'

R; 5'- CCTAATACCACCTACCTATTTCTC -3'

Imprinting analysis of *IGF2R*

F; 5'- TCAGGGGGATTAACCTGATG -3'

R; 5'- ATTGGAAAAAGGGGAGAGAAATC -3'

DNA methylation analysis in the promoter CpG island

Region1F; 5'- AGAGTATTGATGGGTATTTGAGAG -3'

Region1R; 5'- TACCCATTATCACACAAATTAATAC -3'

Region2F; 5'- AAAAGAGGAGGAGGAGGATAG -3'

Region2R; 5'- TAAAACCTCCTAAAATACCC -3'

Region3F; 5'- GTAGTTGTTTTGGAAGTAGGTTTT -3'

Region3R; 5'- TTTTCCTCCTTCCCTCCCTTTCC -3'

Region4F; 5'- TGATAGTTATAAGTGGTTAGGGTTT -3'

Region4R; 5'- AAACCTACTTCCAAAACAACTAC -3'

Initial detection of *ALID* by strand-specific RT-PCR

RT; 5'- ACTCAAAATTCAGCTTGTCC -3'

DetectionF; 5'- TACTGCTACATACGTACACC -3'

DetectionR; 5'- ATCTGCATGTGTATCTTTCC -3'

5' RACE

First; 5'- CTTGCCTCTCTCCAACTCTCGTCCCCTG -3'

Nested; 5'- ACGGGCTACAGGGAAAACACGGACTGC -3'

3' RACE

First; 5'- CCGGACGCTAGGCTTGGCTTCAAGTC -3'

Nested; 5'- GGCTCCTGGGGGTCCACATCAGTATC -3'

Expression analysis of *ALID* by strand-specific RT-PCR

RT; 5'- GGAAAGATACACATGCAGAT -3'

DetectionF; 5'- CCACATCAGTATCCCCAAAG -3'

DetectionR; 5'- CATGCGAAAGGCAGGTAG -3'

Allelic DNA methylation analysis of *ALID*

F; 5'- AGATATTATTATGGTTATTTATGTATTT -3'

R; 5'- CTAAACTTAACTTCAAATCTACTTT -3'
